# Prevalence and Predictors of Internet Addiction Among Adolescents Before the First Wave of COVID-19 Lockdown in India

**DOI:** 10.7759/cureus.59803

**Published:** 2024-05-07

**Authors:** Poornima Narayanappa, Abhay Nirgude, Prasanthi Nattala, Mariyamma Philip, Karthick Subramanian

**Affiliations:** 1 College of Nursing, National Institute of Mental Health and Neurosciences, Bengaluru, IND; 2 Department of Community Medicine, Faculty of Medicine, Yenepoya Medical College, Yenepoya (Deemed to be University), Mangalore, IND; 3 Department of Nursing, National Institute of Mental Health and Neurosciences, Bengaluru, IND; 4 Department of Biostatistics, National Institute of Mental Health and Neurosciences, Bengaluru, IND; 5 Department of Psychiatry, Mahatma Gandhi Medical College and Research Institute, Sri Balaji Vidyapeeth (Deemed to be University), Pondicherry, IND

**Keywords:** india, time before the first wave of covid-19 lockdown, psychosocial predictors, adolescents, internet addiction (ia)

## Abstract

Background

Internet dependency behavior was found to be prevalent among adolescents even before the first wave of COVID-19 lockdowns across the world including India. Adolescent users develop Internet addiction due to various risk factors.

Aim

The aim is to measure the prevalence and psychosocial predictors of internet addiction among adolescents before the first wave of the COVID-19 lockdown in India.

Methods

A cross-sectional, descriptive study before the first wave of the COVID-19 lockdown, included 1199 adolescents of both genders, aged 11 to 19 years, at selected educational settings from a city in south India, by using Young's Internet addiction test (IAT)-20 and structured questioner.

Results

The study found almost all the participants (100%) were using the internet in a day and the highest number of subjects started using the internet during their 6^th^ standard of education (13%). Before the first wave of COVID-19 lockdown, the prevalence of a total of mild, moderate, and severe forms of internet addiction among adolescents was 65%. Individual, family, and community-related risk factors were found significant association with Internet addiction. The age of 14-16 years (OR 2.050, p= 0.000), duration of internet use in a day (OR 0.740, p= 0.064), financial matters (OR 0.981, p=0.016), total internet addiction score (OR 1.03, p=0.035) and timings of internet use (OR 1.161, p=0.004), were significant predictors of Internet addiction.

Conclusion

Internet addiction was prevalent and a notable behavior addiction among adolescents during the margin time of pre-pandemic and the first wave of the COVID-19 lockdown in India. The study highlighted many significant psychosocial risk factors and predictors of Internet Addiction in adolescents, thus the need for a panoramic approach to identify internet addiction in adolescents, to bring the modest behavior of healthy use of the internet in adolescents.

## Introduction

Internet addiction (IA) has been identified as cognitive and behavioral addiction with features of salience, craving, tolerance, withdrawal, and relapse to restart browsing the aforementioned internet activity [1,2]. Availability of the internet, mode of accessibility, browser speed, duration of use, and its purpose were found to influence the uncontrolled use of the Internet [3]. The pre-COVID-19 pandemic time has witnessed an increase in the prevalence of IA among adolescents [4]. These pre-pandemic years have recorded prevalence rates of generalized IA ranging from 12.6% to 67.5% among some European and southeast Asian countries [3], a survey in China noted 23.5% [5] and few studies in India recorded prevalence of severe IA as 3.96% to 4.8% [6,7], Similar observations were evident among Indian adolescents, even after frequent lockdowns of COVID-19 pandemic, for an instance, a study found prevalence of 33% of IA specific to mobile device [8,9]. A good number of studies carried out before the first wave of COVID-19 lockdown identified multiple predictors of IA, which included wide ranges of psychosocial cues, such as internet media, devices, adolescent users, family, parents, peers, and community. A study found the early introduction of a screen to young children, parental style, and models on the liberal use of the Internet at home were important cues to the development of IA in adolescents [10], Peer modeling on the use of the Internet, school environment, and social pressure were instrumental to develop the IA behavior in adolescents, divergent to high expectations from parents, low academic performance and his substance use behavior may influence adolescents to develop IA [11]. Another observation pointed out that the surplus socioeconomic and educational facilities and the adolescents from urban localities were found at risk of developing IA [12]. Additionally, another study identified the blocked communication between parents and teenagers, parental reactions, or heavy restrictions on internet use to provoke curiosity and compulsive internet use in adolescents [13], notable attributing psychosocial predictors such as an increase in screen time, lack of physical socialization make the adolescents to become extremely prone for IA [11,14-16]. The present study investigated the prevalence of IA and its predictors among adolescents before the first wave of the COVID-19 lockdown in India. 

## Materials and methods

Methodology

Study Setting, Design, and Participants

This cross sectional, descriptive study was at a smart city in southern India, with population around 736,000 in which 10% were students, carried out between December 2019 and March 2020, before the lockdown for first wave of the COVID-19 pandemic in India. The participants were school (upper primary and secondary schools) and college students (pre-university, polytechnic diplomas, and first-year undergraduate classes), aged 11 to 19 years, studying in various public and private academic institutions.

Selection of Subjects

The participants were selected through multistage cluster sampling and recruited from two schools and four colleges of the city with any degree of recreational use of internet, irrespective of time/setting/device served as the main inclusion criteria. Students unwilling to participate, children without parental consent, and those who were absent during data collection were not part of the study.

Determining the Sample Size

Power analysis determined the sample size, by keeping prevalence assumption [17] of IA as 24.2%, Absolute precision of 3, Power of 80% and with non-responsiveness of 10 or 20%, Level of significance =5% with 95% confidence level Z=1.96. thus at least 900 subjects were requested to take part in the study. Eventually, due to the high response rate, a total of 1199 adolescents participated in the study.

Study instruments

Assessment of Psychosocial Cues and Internet Usage Patterns

A semi-structured tool for assessing the psychosocial cues and patterns of internet usage in families and among adolescents was constructed by the researchers. Various sources of information such as newspaper reports, scientific publications, and formal and informal communications with subject experts were utilized before the development of the questionnaire. Items such as ownership of a gadget, availability of nonstop internet, duration of use of internet in a day, use of the internet for non-study activity, preference for communication, group or co-browsing, post-usage guilty feelings, fear of developing IA, etc., were some of the elements used in the questionnaire.

Assessment of Family Modeling Patterns

The family modeling patterns in the semi-structured questionnaire included elements such as ‘arguing parents, regular parental supervision, parental attitudes towards gadget use, time restrictions on gadget use, etc.

IA Tool (IAT-20)

The IAT-20 is a self-administered tool that assesses the presence of IA. It has 20 items given rates on a Likert scale from 1 to 5, yielding a maximum score of 100. A score below 30 is considered “normal levels” of internet usage, whereas scores 31-100 are categorized as moderate to severe addiction. Good internal consistency of Cronbach’s α 0.90 was found in various studies with IAT-20 tool in India [18].

Study procedure

The participants were approached at their place of study after finalizing the date and time with the school/college authorities. Information about the study was provided to the participants in English and Kannada, and doubts were clarified by the investigators. Students who assented to study participation were provided consent forms to be filled out by their parents. Only those who provided parent's consent were finally included in the study later. The classroom had 30-40 students at one time and the data was collected without disturbing their academic schedule. The students were given the choice of not filling up the questionnaire and leaving the hall without giving any reason. Appropriate assent/consent and written permissions were obtained from the study participants, parents, and local civil authorities, The study was approved by the institutional ethics committee. Confidentiality and anonymity were maintained across the study and reporting of the study results. 

Statistical analysis

Data analysis was done using SPSS version 28 (IBM Corp., Armonk, NY, USA). Descriptive statistics were carried out to calculate frequencies and percentages for sociodemographic characteristics and psychosocial cues. IAT scores were analyzed as per the recommended guidelines [19]. With univariate analysis, the significant factors which were resulting the prevalence of IA were again loaded multivariate logistic regression analysis. p < 0.05 level was considered statistical significance.

## Results

A total of 1,199 adolescents participated in the study. The current study found that 35% of adolescents used the internet within normal levels, and IA was noted in varying severity among the rest (65%) of the study participants: mild (39%), moderate (23%), and severe (3%). Three participants did not complete the IAT tool.

Sociodemographic characteristics

Most of the adolescents belonged to nuclear families (60%) and resided with their parents (86%), male subjects were in the majority (59%), age 14-16 years (35%), and were studying in secondary classes (42%). In our sample, adolescents with IA more often belonged to the age group of 14-16 years and belonged to 8th to 10th classes. There were no other differences between the subjects with the normal use of the two groups based on other sociodemographic characteristics (Table 1).

**Table 1:** Relationship between demographic variables and Internet addiction (IA) in adolescents *P value significant. ^#^IA=Internet addiction

Variables	Categories	No addiction	Mild-to Severe IA^#^	Chi-Square value (p-value)
Age	11-13 years	148 (36%)	225 (29%)	27.285 (0.001)*
	14-16 years	106 (26%)	319 (41%)	
	17-19 years	160 (39%)	238 (30%)	
Gender	Male	235 (57%)	472 (60%)	1.447 (0.229)
	Female	179 (43%)	310 (40%)	
Class of studying	6-7 ^th^ class	116 (28%)	161 (20%)	23.232 (0.001)*
	8-10 ^th ^class	134 (32%)	364 (47%)	
	PUC and Diploma	98 (24%)	148 (19%)	
	First degree	66 (16%)	109 (14%)	
Type of family	Single parents and children	25 (6%)	26 (3%)	0.370 (0.543)
	Both parents and children	235 (57%)	479 (61%)	
	Grandparents with parents and children	101 (24%)	191 (24%)	
	Grandparents with extended family	53 (13%)	86 (11%)	
Occupation of Father	Unskilled	70 (18%)	123 (17%)	2.064 (0.724)
	Skilled	125 (34%)	248 (34%)	
	Clerical	34 (98%)	81 (11%)	
	Business	98 (57%)	173 (23%)	
	Professional	57 (38%)	114 (15%)	
Occupation of Mother	Employed	201 (49%)	380 (49%)	0.001 (0.989)
	Housewife	207 (51%)	392 (51%)	
Education of Father	Schooling	149 (39%)	267 (36%)	1.147 (0.564)
	PUC/Pre-degree	87 (23%)	169 (23%)	
	Graduation /Post-graduation	142 38%	297 (41%)	
Education of mother	Schooling	145 (37%)	251 (34%)	3.104 (0.212)
	PUC/Pre-degree	78 (20%)	178 (24%)	
	Graduation /Post-graduation	172 (44%)	302 (41%)	
Single child in the family	Yes	95 (23%)	157(20%)	1.341 (0.247)
	No	319 (77%)	625 (80%)	

Influence of psychosocial cues on IA

To understand the psychosocial cues on IA, a set of influencing psychosocial factors was compared between subjects who manifested mild to severe IA (IA+) and those without internet addiction (IA-). Compared to the IA- group, those with IA used the internet for more than two hours every day (p=0.001), often used a smartphone for internet access (p=0.028), felt prouder at having online friends (p=0.006), more often discussed social media and app updates (p=0.005), suffered post-usage guilt (p=0.006), and were more fearful of becoming addicted to the internet (p=0.002). There were no differences between the two groups in other parameters such as ownership of gadgets, internet connectivity, usage of the internet for reasons other than studies, etc., as seen in Table 2.

**Table 2:** Psychosocial cues with internet addiction in study participants (N=1,199) *P-value significant. ^#^IA=Internet addiction

Variables	Categories	No addiction (n=414)	Mild to Severe IA (n=742)	Chi-Square value (p-value)
Ownership of gadget	Uses Own gadget	187 (45%)	324 (41%)	1.852 (0.396)
	Uses family members' gadgets	216 (52%)	440 (56%)	
	Both whichever convenient	11 (3%)	18 (2%)	
Availability of Nonstop internet	Yes	129(31%)	254 (33%)	0.217(0.641)
	No	285(70%)	528 (68%)	
Duration of use of the internet in a day in the past 6 months and more	Less than 1 hour	167 (41%)	354 (45%)	14.88 (0.001)*
	1-2 hours	169 (41%)	235 (30%)	
	More than 2 hrs	78 (19%)	193 (25%)	
Use of the Internet for non-study activity	Yes	172(42%)	316(40%)	0.15 (0.704)
	No	242(59%)	466 (60%)	
Prefers internet-assisted (virtual) communication instead of face-to-face (in-person) communication	No	227(55%)	449(57%)	2.16 (0.340)
	Occasionally	141(34%)	235 (30%)	
	Always	46 (11%)	98 (13%)	
Co-browsing/group browsing	No	156 (38%)	317 (41%)	4.35 (0.114)
	Occasionally	204 (49%)	339 (43%)	
	Always	54 (13%)	126 (16%)	
Believes that the internet provides solutions to all doubts and problems	No	116 (28%)	210 (27%)	3.13 (0.209)
	Occasionally	119 (48%)	348 (45%)	
	Always	99 (24%)	224(29%)	
Internet is accessed on mobile phones (Smartphones)	No	21 (5%)	73 (9%)	7.16 (0.028)*
	Occasionally	104 (25%)	176 (23%)	
	Always	289 (70%)	533 (68%)	
Feel proud of the number of online friends	No	206(50%)	348(45%)	10.29(0.006)*
	occasionally	150(36%)	265(34%)	
	Always	58(14%)	169(27%)	
Follows new trends and fashions with internet gadgets.	No	146 (35%)	284 (36%)	20.52 (0.001)*
	occasionally	183 (44%)	256 (33%)	
	Always	85(21%)	242(31%)	
Proceeds with discussion on internet updates or social media with friends and other people	No	189 (46%)	334 (43%)	10.68 (0.005)*
	occasionally	187 (45%)	323 (42%)	
	Always	38 (9%)	125 (16%)	
Feels sorry for wasting time and energy on internet activities like games, social media, video watching	No	100 (24%)	209 (27%)	10.37 (0.006)*
	Occasionally	224 (54%)	350 (45%)	
	Always	90 (22%)	223(29%)	
Fear of getting addicted to the internet.	No	221 (53%)	376 (48%)	12.85 (0.002)*
	occasionally	120 (29%)	196 (25%)	
	Always	73 (18%)	210 (27%)	

Family-related cues and IA

To ascertain the influence of family on IA, various family-related psychosocial cues for IA were compared between those who had mild to severe IA (IA+) and those without (IA-). Compared to the IA- group, individuals with IA had parents who never kept any time restrictions for mobile use (p=0.010), had parents who opined that “elders can use gadgets as they wish” (p=0.021), and more often noticed that “some family member is very secretive about gadget/internet use” (p=0.052). In contrast, adolescents in the IA- group had parents who more often supervised their daily activities at home (p=0.037) and were worried about their internet or gadget usage (p=0.005) as depicted in Table 3.

**Table 3:** Family model of psychosocial cues of internet addiction P-value significant. ^#^IA=Internet addiction.

Variables	Categories	No addiction (n=414)	Mild-severe IA^#^ (n=742)	Chi-Square value (p-value)
		No: 1996 (100%)		
Internet use for non-academic activity among family members	All are using	235 (57%)	459 (59%)	3.85(0.146)
	Parents only using	75 (18%)	163 (21%)	
	Children only using	104 (25%)	160 (20.5%)	
Parents argue a lot for some reason which you do not like	Yes	147 (36%)	312 (40%)	2.21 (0.137)
	No	267 (65%)	470 (60.1%)	
Parents supervise the daily activities of adolescents at home.	No	50 (12%)	92 (11.8%)	6.58 (0.037)*
	Occasionally	221 (53%)	362 (46%)	
	Always	143 (35%)	328 (42%)	
Parents have the opinion that elders can use gadgets as they wish.	No	250 (60%)	426 (55%)	7.72 (0.021)*
	Occasionally	103 (25%)	190 (24%)	
	Always	61 (14.7%)	166 (21.2%)	
Notices that some family members are very secretive about their gadgets/internet activity	No	293 (70.8%)	502 (64%)	5.91 (0.052)*
	Occasionally	75 (18.1%)	161 (21%)	
	Always	46 (11.1%)	119 (15%)	
Parents keep time restrictions to use internet gadgets like mobile	No	107 (25.8%)	266 (34%)	9.31 (0.010)*
	Occasionally	162 (39.1%)	255 (33%)	
	Always	145 (35%)	261 (33%)	
Parents will enquire about mobile or computer activities.	No	139 (33.6%)	293 (38%)	1.799 (0.407)
	Occasionally	164 (39.6%)	289 (37%)	
	Always	111 (26.8%)	200 (26%)	
Family Prefers to do routine transactions on the Internet.	No	194 (46.9%)	340 (44%)	1.37 (0.503)
	Occasionally	164 (39.6%)	335 (43%)	
	Always	56 (13.5%)	107 (14%)	
Parents are worried about internet /gadget use.	No	165 (39.9%)	292 (37%)	10.46 (0.005)*
	Occasionally	165 (39.9%)	265 (34%	
	Always	84 (20.3%)	224 (28%)	

Predictors of IA in adolescents

The present study found significant predictors such as 'the age of the participant, duration of internet use in a day, financial domain, total IA score and internet use timings for IA among adolescents. The predictors such as the age of 14-16 yrs (OR 2.050, p=0.000) showed adolescents are at higher risk of IA, compared to 17+ age(OR 0.813, p=0.273) and they have less risk of developing IA than younger participants such as 13-14 years old, duration of internet use in a day (OR 0.740, p=0.064), financial matters of the family (OR 0.981, p=0.016), total IA score (OR 1.03, p=0.035)and timings of internet use such as more the number of times of use (before sleep, while sleeping, while studying, while eating, etc.) (OR 1.161, p=0.004), more chance of getting into IA. The logistic regression model had an overall accuracy rate of 66.1 and sensitivity rate of 92.5 and Nagalkerke’s R2 =0.078. this means about 66% of the variation in the outcome variable (internet normal users and IA) could be explained by these predictors (Table 4).

**Table 4:** Predictors of Internet addiction in adolescents *P value significant. ^#^IA=Internet addiction. Variables entered*:* Father's occupation, Education father, the duration of your internet use for non-academic purposes in a day since past six months, age, class of studying, education of mother, family type, occupation with indefinite timings for father and mother, single child in family, non-stop internet available at all time of the day, occupation of mother, parents always argue for some reasons in the family which you do not like.

Significant predictors of IA^#^	OR (95%CI)	P-value
14-16yrs of Age	2.050 (1.476-2.484)	0.000
17-19 years of age	0.813 (0.561-1.177)	0.273
1-2 hrs of the duration of internet use for non-academic activities in a day for the past 6 months.	0.740 (0.539-1.018)	0.064
More than 2 hrs of duration of internet use for non-academic activities in a day the past 6 months	1.242 (0.800-1.926)	0.334
Financial matters of the family	0.981 (0.966-0.997)	0.016
Total internet scores	1.038 (1.003-1.075)	0.035
Timings of your Internet use	1.161 (1.045-1.284)	0.004

## Discussion

The present study found that 65% of study participants have either mild, moderate, or severe levels of IA, in which IA was more frequent among adolescents of 14-16 years. The findings are like those reported in studies before the first wave of the COVID-19 lockdown in India [20,21]. However, IA is constantly found to increase among all age groups adolescents when the observations made in some studies later to social restrictions due to COVID-19 lockdowns in India [22-24]. This could be due to those adolescents who acquired access to smartphones during the pandemic period citing academic reasons but using them for social media and gaming activities [25-27].

In the present study, IA was found in all grades of adolescent students but was higher among high school (8th to 10th class) compared to lower or higher classes. Among adolescents in the group IA, the study found the duration of internet use was more than two hours a day and the internet access were through smartphones. These adolescents felt prouder about having online friends, they like to try out and follow new trends with internet gadgets and even these adolescents felt, they may get addicted to the internet. All these significant risk factors were observed in multi-centered studies across the world [10-12] and contributed to identifying the emerging causes of IA in adolescents during pre-pandemic time, e.g., one study identified 4.8% of severe IA among adolescents of 13-18 years of age at Indian urban setting, the internet use duration was for more than 5 hours a day, the internet was used for non-educational activities and the mobile gadget was used to access internet in the participants [7]. Another study identified adolescents were vulnerable to developing IA with risk factors like longer online hours like 8-10 hours a day, spending virtual-based leisure time, online games, active social media, and watching reels [26].

The family and parental cues in the present study identified three significant risk factors and two important protects against IA in adolescents. Risk factors include the liberal attitude of parents ‘to not to keep any time restrictions for mobile use’ that may lead to the development of internet use gratifications in adolescents. Parents of adolescents in the IA group believed that only elders can use the gadgets as they wish but not adolescents might have triggered the urge to use the gadgets, another risk factor depicted adolescents' observation of some of their family members who are secretive about internet or gadget usage may provoke the curiosity to explore. The protective factors of the no-IA group were those adolescents perceived that their parents were supervising their daily activities at home and their parents were worried about gadget use by their wards. At the outset, researchers feel both factors were acceptable by adolescents in the Indian family context. Supportive evidence from studies, before the first lockdown of the COVID-19 pandemic also depicted the vital role of parents and family in IA in adolescents. A study identified parenting style and ignorance of the mother, inconsistent upbringing of children and not monitoring the online activities of children were significant risk factors for developing IA in 10-18-year-old adolescents [5]. Another study identified lower family functioning and reduced interpersonal trust as a risk factor for IA in adolescents and those adolescents were prone to depression [26]. Moreover, the present study identified the possible predictors such as ‘age, duration of internet use in a day, financial domain, internet use timings, and total IA score’ among internet normal users and adolescents with IA before the first lockdowns of the COVID-19 pandemic in India. Similar observations were made on the age of the internet user, timings of browsing, and influence of parental use of the internet or gadget as important predictors [27,28]. Yet another review identified unjustified internet use restrictions from parents, and IA among some family members were 'triggers' but nicely educated parents were ‘protects’ against IA in adolescents [29], In addition to these, a study identified neurotic and introverted personality traits, as well as maladaptive cognitions, played the role of predictors of IA in adolescents and protectors were personality types of adolescents such as extraversion, openness, agreeableness, and conscientiousness [30].

Limitations: This research had some limitations. First, the study did not measure any specific types such as smartphone addiction, video-online games, social media, or pornography addiction, instead, the overall or aggregate recreational use of the internet in adolescents was measured to know the prevalence and predictors of IA, If done, the study might have measured the specific types of IA which is not in the purview of the study. Secondly, the study identified attributable psychosocial predictors and risk factors of IA, but it failed to establish definite causal relationships between study variables and IA. Lastly, despite assuring the anonymity and confidentiality of the research, adolescents might have experienced the fear of threat to their autonomy about using the internet facilities/gadgets, which might have influenced subjects to probable masking of actual information.

## Conclusions

Findings of the present study concluded that overall, 65% of the prevalence of IA was found either in mild, moderate, or severe forms among Indian adolescents during the pre-pandemic time before the first wave of the COVID-19 lockdown. The study identified the age of 14-16 years, high school studying adolescents were affected more among adolescents of age 11 to 19 years. The study identified adolescents who were using the Internet for more than 2 hours a day, who used smartphones to access the Internet, who felt prouder for having more online friends, who would like to try out and follow new trends with Internet gadgets and who feared themselves for getting addicted were in IA group. The present study ascertained the family and parents related risk factors such as passive parenting “not to keep any time restrictions for mobile use” parents believing that elders can use gadgets as they wish, and “some of the family members are secretive about internet or gadget usage were influencing the IA in adolescents.” The study recognized two protective factors against IA namely regular supervision of daily activities by parents at home and the perception of adolescents on their parents who were worried about gadget use by their children. 

Frequent and exaggerated use of recreational use of the internet and gadgets may cause IA in the vulnerable age group of adolescents. The present study identified predictors such as “age, duration of internet use in a day, financial domain, internet use timings, and total IA score” with IA in adolescents before the time of the first lockdown of the COVID-19 pandemic. Thus parents and concerned society people need to understand the importance of protecting adolescents against IA to preserve the mental health of adolescents. Age-appropriate internet safe use measures can be taught to adolescents at family and schools. The present study findings also urge for the implementation of systematic actions by family and parents as well as adolescents that are crucial to prevent IA in adolescents.
